# Identification of differentially methylated genes in the malignant transformation of ovarian endometriosis

**DOI:** 10.1186/1757-2215-7-73

**Published:** 2014-07-10

**Authors:** Fang Ren, Dan-Bo Wang, Tong Li, Ying-Han Chen, Yan Li

**Affiliations:** 1 Department of Obstetrics and Gynecology, Shengjing Hospital of China Medical University, 36 Sanhao Street, Shenyang 110004, China

**Keywords:** Ovarian carcinoma, Endometriosis, Malignant transformation, Methylation, RASSF2

## Abstract

**Background:**

Key roles for epigenetic mechanisms in tumorigenesis are well accepted, while the relationship between gene methylation and malignant transformation of ovarian endometriosis (EMS) was seldom reported. In this study, we aimed to screen for aberrantly methylated genes associated with the malignant transformation of ovarian EMS and to preliminarily verify the reliability of screened results by detecting the methylation status and protein expression of the candidate gene in a larger scale of formaldehyde-fixed and paraffin-embedded (FFPE) samples.

**Methods:**

Methylated CpG island amplification coupled with representational difference analysis (MCA-RDA) was performed on 3 couples of endometriosis-associated ovarian carcinoma (EAOC) fresh samples to identify differentially methylated candidate genes related to malignant transformation of ovarian EMS; Methylation-specific PCR (MSP) and immunohistochemistry were performed in 30 EAOC samples to detected the methylation status and protein expression of RASSF2 gene to verify the reliability of MCA-RDA results.

**Results:**

Nine differentially methylated genes were obtained by MCA-RDA as candidate genes for malignant transformation of EMS; Methylation frequency of RASSF2 in the neoplastic tissues of EAOC group was higher than that in the ectopic endometria (p < 0.05). While protein expression of RASSF2 in the neoplastic tissues was lower than that in the ectopic endometria of the EAOC group (p < 0.05) Absence of protein expression of RASSF2 was significantly correlated with the promoter methylation of the gene (p < 0.05).

**Conclusions:**

RASSF2, RUNX3, GSTZ1, CYP2A, GBGT1, NDUFS1, SPOCK2, ADAM22, and TRIM36 were candidate genes for malignant transformation of ovarian EMS and epigenetic inactivation of RASSF2 by promoter hypermethylation is an early event in malignant transformation of ovarian EMS. The screen results were reliable and worthy of further study.

## Background

Endometriosis (EMS) is a common and refractory gynecological disease that affects 15% of women of childbearing age. Malignant transformation of EMS is believed to occur in ~1% of all cases of EMS. The most common site of malignant transformation of EMS is the ovary, and this type of ovarian carcinoma is known as EMS-associated ovarian carcinoma (EAOC). Ovarian endometrioid cancer (OEC) and ovarian clear cell cancer (OCCC) account for 76% of all EAOC diagnoses [1]. Ovarian cancer was previously considered to be a primary disease which originated from the ovarian surface epithelium and developed into different subtypes after being stimulated by multi-directional external stimuli, and the relationship between ovarian cancer and endometriosis was confused. Recently,the “dualistic model” hypothesis of the pathogenesis of ovarian cancer has gained favor [2]. The core of the model is the theory that ovarian cancer originates from tissue implantation outside of the ovary, such as ovarian serous adenocarcinoma is thought to originate from tubal fimbria epithelial lesions, OEC and OCCC are likely to originate from EMS, and the ovarian mucinous and transitional cell tumors probably originate from Wathard cell nests next to the ovary. Since then it is widely accepted that most of OEC and OCCC originate from EMS [3]. And investigation of the mechanism of malignant transformation of EMS will supply new ideas for the study on the pathogenesis of these two special types of ovarian cancer. Although the study of malignant transformation of EMS has been a “hot topic” in the research of endometriosis, the degree of progress has remained slow. Generally-accepted diagnostic criteria for the malignant transformation of EMS were proposed by Sampson [4] and Scott [5]. However, obliteration of the preexisting EMS by the overgrown tumor or failure to sample the minute foci of EMS has resulted in missed diagnoses and insufficient numbers of samples for preliminary investigation. In addition, the polymorphic pathological features of EMS have made it difficult to accurately obtain the ectopic endometrium. The extent of basic research into EMS malignant transformation is far less complete as compared to that of other types of tumors.

Aberrant DNA methylation is an important epigenetic alteration that is intimately involved in the development of human tumors, aberrant methylation of CpG islands in the promoter is known to be a major inactivation mechanism for tumor-related genes. At present, there are reports of aberrant methylation of candidate genes related to malignant transformation of EMS [6,7], however, specific genes have not yet been identified. Methylated CpG island amplification coupled with representational difference analysis (MCA-RDA) is a rapid high-throughput methylation spectral analysis technique involving a subtraction hybridization-based assay that allows for the rapid amplification and selection of densely methylated CpG-rich regions, MCA-RDA can be used to identify differentially-methylated CpG islands between two types of tissues. The advantage of this technique is that it can not only find the known methylated fragments, but can also identify previously unknown methylated fragments. The MCA-RDA is limited by the requirement for high quality of DNA samples, as the integrity of the DNA is destroyed during the process of formaldehyde-fixed and paraffin-embedded (FFPE), generally the FFPE sample cannot be used [8]. In this study, having obtained fresh tissue samples from three patients with EAOC, we performed MCA-RDA analysis to identify differentially-methylated candidate genes related to the malignant transformation of ovarian EMS. After detecting the methylation status and protein expression of the candidate gene, RASSF2, in a larger scale of FFPE samples to preliminarily verify the reliability of MCA-RDA results.

## Materials and methods

### Tissue samples

All specimens were obtained from patients hospitalized in the Department of Obstetrics and Gynecology of Shengjing Hospital (Shenyang, China). For the EAOC group, three fresh tissue samples (one OEC sample and two OCCC samples) were obtained at the time of surgery and used for MCA-RDA; 30 FFPE tissue specimens (18 OEC and 12 OCCC) were obtained from patients who were diagnosed between January 2005 and January 2012 and used to measure the methylation status and protein expression of the candidate gene (21 cases for Stage I and 9 cases for Stage II). Specimens were reviewed by a gynecologic pathologist to confirm the histopathologic diagnosis according to Sampson and Scott’s criteria. Thirty 30 ovarian EMS specimens were collected as the EMS group and 20 normal endometrial specimens were collect as the normal endometrium (NE) group. Informed consent was obtained from all patients or their family members, and agreement was obtained from the Ethics Committee of China Medical University before collecting the samples. The average age of the patients in the three groups were 44.19 ± 9.86 ,43.12 ± 4.20 and 43.52 ± 5.16 years, respectively; there was no statistical difference in age among the three groups (p > 0.05). None of the patients had received any GnRH analog or other hormonal medication or antibiotics in the six months prior to the surgery.

### Screening the candidate genes by MCA-RDA

Fresh EAOC specimens from three patients were fast-frozen. Then, the frozen tissue was cut into 10-μm sections and the malignant tissues and the ectopic endometrial tissues were obtained by microdissection. Genomic DNA was extracted from the target tissues using standard phenol-chloroform methods. The three malignant tissues were mixed and used as the “tester”, while three paired ectopic endometrial tissues adjacent to the cancerous region were mixed and used as the “driver”. MCA-RDA was performed as described previously [9]. Briefly, 5 μg of genomic DNA for both the tester and driver were digested with SmaI (New England Biolabs, Beverly, MA) for 16 h followed by XmaI (New England Biolabs, Beverly, MA) for 8 h. The restriction fragments were ligated to the RXMA 24/12 adapter using T4 DNA ligase (New England Biolabs, Beverly, MA) and methylated CpG islands were amplified by polymerase chain reaction with the RXMA24 primer. Then, the adaptor of the ‘driver’ was cut off using the SmaI restriction enzyme, and the adaptor of the ‘tester’ was cut off using the XmaI restriction enzyme, and new adaptors were added. The tester and driver underwent three cycles of hybridization RDA analysis at ratios of 1:80, 1:160, and 1:320. After each cycle, the adaptors were changed. The adaptors used in the three cycles of analysis were RXMA24/12, JXMA24/12, and NMCA24/12, respectively (primers shown in Table 1). The RDA products were ligated to vectors (Promega, Madison, WI) using T4 ligase and then transformed into DH5α competent *E.coli* bacteria for incubation in matrix containing ampicillin. A total of 96 positive clones were obtained and sequenced. Sequences were analyzed using Blast (http://www.ncbi.nlm.nih.gov/BLAST/) and BLAT (http://genome.ucsc.edu).

**Table 1:** Primers used in this study

**Name**	**Sequence**
**MCA-RDA primers**	
RXMA24	5’-AGCACTCTCCAGCCTCTCACCGAC-3’
RXMA12	5’-CCGGGTCGGTGA-3’
JXMA24	5’-ACCGACGTCGACTATCCATGAACC-3’
JXMA12	5’-CCGGGGTTCATG -3’
NMCA24	5’-GTTAGCGGACACAGGGCGGGTCAC-3’
NMCA12	5’-CCGGGTGACCCG-3’
**MSP primers for RASSF2**	
RASSF2 MF	5’-GTTCGTCGTCGTTTTTTAGGCG-3’
RASSF2 MR	5’-AAAAACCAACGACCCCCGCG-3’
RASSF2 UF	5’-AGTTTGTTGTTGTTTTTTAGGTGG-3’
RASSF2 UR	5’-AAAAAACCAACAACCCCCACA-3’

### Preliminary verification of the reliability of MCA-RDA results

FFPE specimens were serially cut into 10-μm sections and microdissection was performed under a dissecting microscope with a 21-gauge needle. Since no typical ectopic endometrial tissue was found besides the sample section, ectopic endometrial tissue was failed to be obtained from four cases. Also, due to the pathological polymorphism of the ectopic endometria and the obliteration by the overgrown neoplastic tissue, the amount of the ectopic endometrial tissue obtained from another four cases could not meet the need for experiment. Finally only 22 ectopic endometrial tissue samples adjacent to the cancerous region were obtained from the EAOC group. In addition, 30 malignant tissue and 20 eutopic endometria samples from the EAOC group, 30 ectopic endometrial and 20 eutopic endometrial samples from the EMS group and 20 normal endometrial samples from the NE group were obtained.

Genomic DNA was extracted from target tissue using the QIAamp DNA Micro kit (Qiagen, Hilden, Germany) according to the manufacturer’s protocol. Genomic DNA was modified by sodium bisulfite at 55°C for 16 h, and the modified DNA was purified using Promega wizard DNA purification resin (Promega, Madison, WI). After completed modification by NaOH, the modified DNA was resuspended in water. During the modification process, normal leukocyte DNA treated *in vitro* with or without SssI methyltransferase was used as a positive or negative control for methylated alleles of the gene. The methylation status of RASSF2 was determined by methylation-specific PCR (MSP); methylation-specific and unmethylation-specific primers were used to amplify the RASSF2 gene promoter as shown in Table 1. The PCR reaction products were directly loaded onto 2% agarose gel stained with ethidium bromide and visualized under UV illumination.

The protein expression of RASSF2 was evaluated by immunohistochemistry, which was performed with a standard streptavidin-peroxidase technique using diaminobenzidine as a chromogen. Goat polyclonal antibody against RASSF2 (dilution 1:100; Santa Cruz Biotechnology, Santa Cruz, CA, USA) was used as the primary antibody; PBS was used as the negative control. The normal staining patterns for RASSF2 is cytoplasm. The percentage of cells positive for RASSF2 expression was evaluated by counting at least five different areas of the sections at high magnification (×1000). The immunoreactivity was determined by two independent observers.

### Statistical correlation

Statistical analysis was performed using SPSS11.0 software, the chi-squared test, and Fisher’s exact test, as appropriate. A probability value <0.05 (two-sided) was considered statistically significant.

## Results

### Identification of differentially methylated sequences in the malignant transformation of ovarian EMS

After three rounds of analysis, 96 positive clones containing the MCA-RDA fragments were random selected. The lengths of the MCA-RDA fragments were detected by amplification with SP16 and T7 primers; 68 of the products which were longer than 100 bp were selected to undergo sequencing analysis. Finally, 40 independent differentially methylated sequences were obtained and 14 of them satisfied the CpG island criteria proposed by Takai and Jones [10]. Screening the 14 CpG islands using BLAST and BLAT revealed that nine of them were localized within or in close proximity to the promoter region of genes (Table 2). The corresponding genes thus became candidate genes for the malignant transformation of ovarian EMS. The genes screened in this study were divided into four categories according to their function, including: (1) apoptosis-related genes: RASSF2; (2) metabolism-related enzyme genes such as GSTZ1, CYP2A, GBGT1, and NDUFS1; (3) cell adhesion-related genes such as SPOCK2 and ADAM22; and (4) gene expression regulation-related genes such as RUNX3 and TRIM36. Among nine candidate genes, known tumor-related genes were RASSF2, RUNX3, GSTZ1, CYP2A, NDUFS1, SPOCK2, ADAM22 and TRIM36; CYP2A has been reported to be associated with ovarian cancer, and RASSF2 and RUNX3 have been reported to be associated with endometrial cancer; none of the genes has yet been associated with EMS. Our follow-up experiments of this study confirmed that hypermethylation of SPOCK2 is associated with the malignant transformation of ovarian EMS [11].

**Table 2:** Details of the differentially methylated sequences

**Gene**	**Accession**	**Length**	**Identity**	**Chrosome**	**CpG island**	**TO TSS**
**GBGT1**	NT_035014.4(2815260-2815716)	455	99.8%	9	YES	525
**GSTZ1**	NT_026437.12(58787254-58787552)	285	99.7%	14	YES	28
**SPOCK2**	NT_030059.13(24652170-24652330)	153	98.8%	10	YES	924
**NDUFS1**	NT_005403.17(57233594-57233371)	229	96.6%	2	YES	57
**CYP2A**	NT_011109.16(13910095-13910256)	162	100.0%	19	YES	162
**RUNX3**	NT_004610.19(11936048-11935819)	228	100.0%	1	YES	810
**RASSF2**	NT_011387.8(4743572-4743752)	167	98.9%	20	YES	539
**ADAM22**	NT_007933.15(25596754-25596538)	217	100.0%	7	YES	129
**TRIM36**	NT_034772.6(22829799-22829407)	393	100.0%	5	YES	316

### Verification the relationship between epigenetic silencing of RASSF2 and the malignant transformation of ovarian EMS

**Figure 1:**
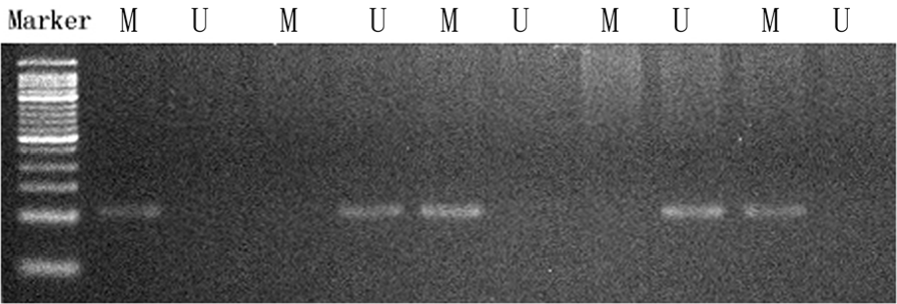
**Methylation-specific PCR (MSP) of RASSF2 in the target tissue.** Marker: 50 bp ladder; M: product of methylation-specific primers; U: product of unmethylation-specific primers.

**Figure 2:**
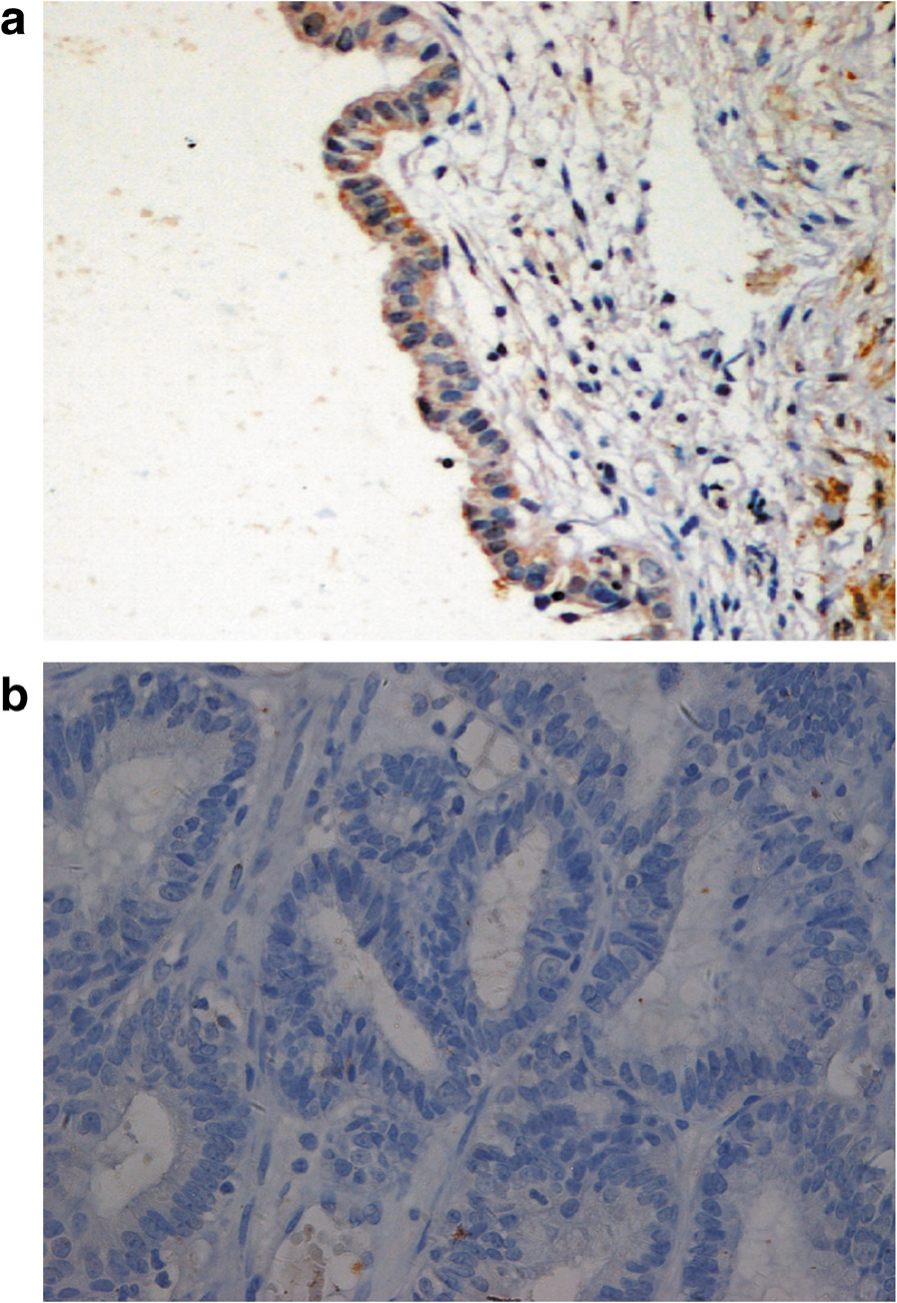
**Protein expression of RASSF2 in the target tissue. a**. Normal RASSF2 protein expression in ectopic endometrium. **b**. Absence of RASSF2 protein expression in endometriosis-associated ovarian endometrioid cancer. Magnification × 200.

Because of the polymorphic pathological features of the ectopic endometrium, it is difficult to obtain the ectopic endometrium from the EMS samples and even more difficult to obtain neoplastic tissue and ectopic endometrium simultaneously from the EAOC samples. In this study, we used microdissection to obtain 22 ectopic endometrial tissue samples adjacent to the cancerous region from 30 EAOC samples; however, as we were limited by the amount of the ectopic endometrium, methylation status and protein expression could be measured for only one candidate gene. RASSF2, which was most frequently repeated during MCA-RDA screening was further studied (shown in Figure 1 and Figure 2), and the relationship between epigenetic inactivation of the remaining genes and EAOC will be detected in other group of EAOC samples in future. The frequency of promoter hypermethylation and protein expression of RASSF2 in the target tissue was shown in Table 3. In the EAOC group, methylation frequency of RASSF2 in the neoplastic tissues was higher than in the ectopic and eutopic endometrial tissues (p < 0.05), and there was no statistical difference of that between the ectopic and eutopic endometrial tissues (p > 0.05); In the EMS group, there was no statistical difference in methylation frequency of RASSF2 between the ectopic and the eutopic endometrial tissues (p > 0.05); There was no statistical difference in the methylation frequency of RASSF2 between the ectopic endometrial tissues of the EAOC group and the EMS group (p > 0.05), and there was also no statistical difference in the methylation frequency of RASSF2 in the eutopic endometrial tissues among three groups (p > 0.05). Meanwhile, protein expression of RASSF2 in the neoplastic tissues was lower than that in the ectopic and eutopic endometrial tissue of the EAOC group (p < 0.05), while there was no statistical difference in the protein expression of RASSF2 between the ectopic endometrial tissues of the EAOC group and the EMS group (p > 0.05). There was no statistical difference in the protein expression of RASSF2 between the ectopic and the eutopic endometrial tissues in both the EMS group and the EAOC group (p > 0.05), and there was no statistical difference in the protein expression of RASSF2 in the eutopic endometrial tissues among three groups (p > 0.05). The ratio of protein expression absence of RASSF2 was 96.3% (26/27) in the RASSF2 methylated tissue. Absence of protein expression of RASSF2 was significantly correlated with the promoter methylation of the gene (p < 0.05).

**Table 3:** Methylation status and protein expression of RASSF2 in the target tissue

	**Methylation**		**Unmethylation**		**Total number**
	*SP(+)	^**#**^**SP(-)**	**SP(+)**	**SP(-)**	
EAOC group					
Neoplastic tissue	1	15	13	1	30
Ectopic endometria	0	3	18	1	22
Eutopic endometria	0	3	17	0	20
EMS group					
Ectopic endometria	0	3	26	1	30
Eutopic endometria	0	1	19	0	20
NE group					
Normal endometria	0	1	19	0	20

*SP(+): RASSF2 protein expression (+); ^#^SP(-): RASSF2 protein expression (-).

## Discussion

Key roles for epigenetic mechanisms in tumorigenesis are well accepted, and the relationship between genes’ methylation and EMS was multiple reported [12], while the relationship between genes’ methylation and malignant transformation of EMS was rarely reported. Martini et al. [6] hypothesized that EMS with a hypermethylated hMLH1 promoter and a consequent absence of gene product could be the malignant precursor of endometrioid carcinoma. This study preliminary reported that gene hypermethylation could play an important role in the malignant transformation of ovarian EMS. Recently, another investigation implicated that LINE-1 hypomethylation was involved in OEC and OCCC malignant transformation [7]. Further investigation is needed to clarify whether such aberrant methylation occurs more widely and affects more genes in the malignant transformation of ovarian EMS. Though techniques for screening aberrant methylated genes have been emerging in the study of pathogenesis of other malignancies, there has been no report as yet describing the screening for aberrant methylation of genes related to EAOC, most likely due to the requirement for fresh tissue specimens.

In this study, fresh tissue samples from three EAOC patients were obtained during the follow-up process for our EMS outpatients managed with conservative treatment therapy; the samples were used to identify differentially methylated candidate genes related to the malignant transformation of ovarian EMS by MCA-RDA. Nine differentially methylated candidate genes were identified as potentially related to the malignant transformation of ovarian EMS. After searching relevant literature related to the nine candidate genes, we found that the functions of the candidate genes included apoptosis, metabolism, cell adhesion, and gene expression regulation, and we hypothesized that aberrant methylation of these candidate genes might be involved in the malignant transformation of ovarian EMS by inhibiting gene transcription and silencing gene expression. We also found that two genes, RASSF2 and RUNX3, were reported to be associated with endometrial carcinoma. Malignant transformation of the ectopic endometrium shares some common risk factors and characteristics with that of eutopic endometrium, such as age, obesity and pathological characteristics. Meanwhile, research has shown that the rate of malignant transformation and histological type of the ectopic endometrium were the same as that of the eutopic endometrium [13], which indicated that there might be relationship between the ectopic and eutopic endometrium in the mechanism of malignant transformation. Since then, the correlation between malignant transformation of EMS and the two candidate genes merits further investigation. Meantime, among the nine candidate genes, there was only one gene, CYP2A, which was reported to be related to ovarian cancer and none of the genes has yet been reported to be associated with EMS. Whether the remaining eight genes were EAOC-specific genes that could be used for the diagnosis and treatment of EAOC is worthy of further study.

Endometriosis is an estrogen-dependent disease and promoter’s methylation of the steroid receptor genes have been reported to relate to many diseases [14]. Study have demonstrated that epigenetic changes occur in both promoter regions B and A of the progesterone receptors (PGR) gene in intestinal EMS [15] and hypomethylation of estrogen receptor (ESR) β promoter might contribute to progesterone resistance in women with EMS [16]. ESR and PGR are quite always absent in the EAOC tissue [17], while we did not identify differentially methylated ESR and PGR genes by MCA-RDA in this study. The reason might be that the ESR and PGR loss their gene expression by other mechanism, such as the increased steroid receptor RNA activator protein (SRAP) [18] instead of gene aberrant methylation. While, in this study, we identify a differentially methylated RUNX3 gene which was reported to be related to ESR. Study had demonstrated that RUNX3 could inhibit the transcriptional activation of ERα and function as a tumor suppressor in the Runx3+/− female mice breast cancer model [19]. While the interaction between RUNX3 and ESR or PGR in the malignant transformation of EMS is unknown and is one of the subjects of our ongoing follow-up studies.

The screening result indicated that gene aberrant hypermethylation was a common epigenetic event in the malignant transformation of ovarian EMS which laid the foundation for the study of the epigenetic mechanisms for the malignant transformation of EMS. To verify the reliability of the screening results, we needed to expand the sample size to verify the relationship between methylation of the nine candidate genes and EAOC, but limited by the amount of target tissue obtained from FFPE tissue, especially the ectopic endometrial tissues, we could choose only one candidate gene to verify the screening results. RASSF2, which was more repetitive during screening was chosen. RASSF2 is a member of the Ras association domain family. It encodes a protein containing a Ras-associated domain and it is a negative effector for K-ras via its interaction with K-ras in a GTP-dependent manner. RASSF2 is a novel tumor-suppressor gene that has been shown to play an important role in the Ras signaling pathway [20]. RASSF2 is widely expressed in humans and its down-regulation caused by aberrant promoter methylation had been found in a variety of primary malignant tumors and tumor cell lines [21,22], but it has not been reported to be related to ovarian cancer. A study on gastric cancer suggested that RASSF2 not only suppressed tumor growth directly but also inhibited inflammation and angiogenesis by inhibiting the Ras-signaling pathway [15]. This study supported that RASSF2 was a negative effector of the Ras signaling pathway. Otsuka et al. [23] reported that up-regulation of the Ras gene by abnormal activation might play an important role in malignant transformation of EMS. In addition, Fauvet et al [24] indicated that activation of K-ras gene which was an oncogene and was involved in the Ras signaling pathway might be a late event involved in the malignant transformation of ovarian EMS. The reports above further support that RASSF2 is a candidate gene for the malignant transformation of ovarian EMS. In the present study, the data revealed that the frequency of RASSF2 promoter hypermethylation was significantly higher and that the protein expression of RASSF2 was significantly lower in the neoplastic tissue than in the ectopic endometrial tissue. These data indicated that epigenetic inactivation of RASSF2 by promoter hypermethylation might play an important role in the malignant transformation of ovarian EMS and preliminarily confirmed the screening results. Meantime, we found that epigenetic inactivation of RASSF2 could be detected in 3 cases of ectopic endometrium adjacent to the cancerous region and the corresponding neoplastic tissue were also epigenetic inactivated, this results verified that epigenetic inactivation of RASSF2 in the ectopic endometrial might be an early event in malignant transformation of ovarian EMS. However, the regulation mechanism of the gene in the malignant transformation of ovarian EMS warrants further investigation.

The pathogenesis of EMS remains debated, but the theory of implantation posits that endometrial cells and fragments refluxed during the menstrual period contribute to the pathogenesis of EMS was widely accepted. The theory gives the support to the homology in the origin between the eutopic and ectopic endometrial. The homology can explain the result that no statistical differences of the epigenetic inactivation frequency of RASSF2 was found between the ectopic and the eutopic endometrial of the EAOC and EMS group, and it also inferred that hypermethylation of RASSF2 might not be involved in the pathogenesis of EMS and only be related to the malignant transformation of EMS.

It should be noted we had further detected the methylation status and protein expression of another candidate gene, SPOCK2, in another group of EAOC samples in the follow-up experiment of this study [11]. In that study, 25 neoplastic tissue samples and 15 ectopic endometrial tissue samples adjacent to the cancerous region were obtained from the EAOC group. The methylation statue of SPOCK2 was determined by combined bisulfate restriction analysis and the protein expression of SPOCK2 was evaluated by immunohistochemistry. The results shown that the frequency of SPOCK2 promoter hypermethylation in neoplastic tissue (13/25) was higher than that in the ectopic endometrium (1/15)(p < 0.05), and promoter hypermethylation of SPOCK2 was significantly correlated with the absent of its protein expression(p < 0.05). The results had confirmed that epigenetic inactivation of SPOCK2 by promoter hypermethylation was also involved in the malignant transformation of ovarian EMS. That study had further confirmed the reliability of the screening results and had retrieved the deficiencies brought by limited amount of specimens in this study.

## Conclusions

In this study, we identified nine differentially methylated genes by MCA-RDA, which might be involved in the malignant transformation of ovarian EMS. Meanwhile, our study confirmed that epigenetic inactivation of RASSF2 was associated with malignant transformation of ovarian EMS and might be an early event in the malignant transformation of ovarian EMS The relationship between the remaining candidate genes and EAOC needs further study. A major limitation of this study is that, limited by the amount of ectopic endometrial tissue available, the relationship between epigenetic inactivation of the remaining screened candidate genes and EAOC was undetectable. We will continue to accumulate pathological specimens of EAOC and further verify the remaining screening results of this study.

## Abbreviations

EMS: Endometriosis; MCA-RDA: Methylated CpG island amplification coupled with representational difference analysis; EAOC: Endometriosis-associated ovarian carcinoma; MSP: Methylation-specific PCR; OEC: Ovarian endometrioid cancer; OCCC: Ovarian clear cell cancer; FFPE: Formaldehyde -fixed and paraffin-embedded; ESR: Estrogen receptor; PGR: Progesterone receptors.

## Competing interests

The authors declare that they have no competing interests.

## Authors’ contributions

FR carried out MCA-RDA analysis to screen the differentially methylated genes and drafted the manuscript. DBW participated in the design of the study and performed the statistical analysis. TL carried out MSP. YHC carried out immunohistochemistry, YL participated in its design and helped to draft the manuscript. All authors read and approved the final manuscript.
